# CaRE@ELLICSR: Effects of a clinically integrated, group‐based, multidimensional cancer rehabilitation program

**DOI:** 10.1002/cam4.7009

**Published:** 2024-03-08

**Authors:** Christian J. Lopez, Daniel Santa Mina, Victoria Tan, Manjula Maganti, Cheryl Pritlove, Lori J. Bernstein, David M. Langelier, Eugene Chang, Jennifer M. Jones

**Affiliations:** ^1^ Department of Supportive Care, Princess Margaret Cancer Centre Toronto Ontario Canada; ^2^ Institute of Medical Science, University of Toronto Toronto Ontario Canada; ^3^ Faculty of Kinesiology and Physical Education University of Toronto Toronto Ontario Canada; ^4^ Department of Anesthesia and Pain Management University Health Network Toronto Ontario Canada; ^5^ Institute of Health Policy, Management and Evaluation, University of Toronto Toronto Ontario Canada; ^6^ Department of Biostatistics, Princess Margaret Cancer Centre Toronto Ontario Canada; ^7^ Applied Health Research Centre, Li Ka Shing Knowledge Institute, St. Michael's Hospital Toronto Ontario Canada; ^8^ Social and Behavioural Health Sciences, Dalla Lana School of Public Health Toronto Ontario Canada; ^9^ Department of Psychiatry University of Toronto Toronto Ontario Canada; ^10^ Present address: Department of Social Policy and Intervention University of Oxford Oxford England

**Keywords:** cancer, exercise, implementation, quality of life, rehabilitation, supportive care, survivorship

## Abstract

**Background:**

Although oncology clinical practice guidelines recognize the need and benefits of exercise, the implementation of these services into cancer care delivery remains limited. We developed and evaluated the impact of a clinically integrated 8‐week exercise and education program (CaRE@ELLICSR).

**Methods:**

We conducted a mixed methods, prospective cohort study to examine the effects of the program. Each week, participants attended a 1‐h exercise class, followed by a 1.5‐h education session. Questionnaires, 6‐min walk tests (6MWT), and grip strength were completed at baseline (T0), 8 weeks (T1), and 20 weeks (T2). Semi‐structured interviews were conducted with a sub‐sample of participants about their experience with the program.

**Results:**

Between September 2017 and February 2020, 277 patients enrolled in the program and 210 consented to participate in the research study. The mean age of participants was 55 years. Participants were mostly female (78%), white/Caucasian (55%) and half had breast cancer (50%). Participants experienced statistical and clinically meaninful improvements from T0 to T1 in disability, 6MWT, grip strength, physical activity, and several cancer‐related symptoms. These outcomes were maintained 3 months after program completion (T2). Qualitative interviews supported these findings and three themes emerged from the interviews: (1) empowerment and control, (2) supervision and internal program support, and (3) external program support.

**Conclusions:**

This study demonstrates the impact of overcoming common organizational barriers to deliver exercise and rehabilitation as part of routine care. CaRE@ELLICSR demonstrated clinically meaningful improvements in patient‐reported and functional outcomes and was considered beneficial and important by participants for their recovery and wellbeing.

## 1 INTRODUCTION

Cancer survivors endure physical, functional, and psychosocial challenges prior to, during and after cancer treatment. Cancer‐related impairments are often undertreated and can result in reduced ability to perform basic and instrumental activities of daily living, recreational activities, and consequently, quality of life.^1^, ^2^ Thus, efforts to support the integration of cancer rehabilitation into oncology practice have become a priority to prevent, minimize, and restore these functional and quality of life losses.^3^, ^4^, ^5^

Comprehensive cancer rehabilitation encompasses the coordinated delivery of interventions by a multidisciplinary team, including physical and occupational therapists, exercise professionals, social workers and dieticians, to name a few.^6^ A multidimensional approach including both physical and psychosocial interventions is recommended to treat and manage the numerous and concurrent impairments people with cancer experience.^7^ These interventions have been shown to be cost‐effective^5^ and mitigate the costs associated with reduced work productivity and early retirement.^8^, ^9^ Multidimensional cancer rehabilitation interventions typically include exercise and education. Strong evidence demonstrates exercise provides wide‐ranging health benefits for cancer survivors, including reduced fatigue, improved physical function, and improved quality of life.^10^, ^11^, ^12^ Additionally, self‐management educational interventions centerd on improving patients' knowledge, skills, and confidence with managing cancer‐related impairments have the potential to improve various symptoms and quality of life.^13^, ^14^, ^15^, ^16^ However, the implementation of multidimensional cancer rehabilitation programs into real‐world practice is limited.

Despite clinical practice guidelines defining exercise and education as core components of survivorship care,^7^ they are sparsely implemented into routine clinical practice. Further, evidence of their effect in practice is limited. Accordingly, our team developed and implemented an 8‐week exercise and education program to be delivered as part of routine care.

We sought to overcome commonly reported organizational barriers to implementing exercise and rehabilitation programming into routine cancer care (e.g., inadequate infrastructure to support the delivery of exercise, absence of established referral pathways and networks, and limited resources to deliver the program).^17^ First, funding was obtained to build the infrastructure needed for the program as well as to deliver the program free to participants. Second, the team conducted a series of presentations and case studies at disease site rounds to increase awareness of the program and receive feedback from clinicians on efficient and well‐organized referral pathways that could be developed. For instance, a brief and user‐friendly referral form was developed that was embedded into the centre's electronic medical record with auto‐fax to facilitate referrals to the program. Additionally, oncologists refer patients to a multidisciplinary rehabilitation staff at the cancer center to further assess the patient and refer to the program when appropriate. This process aimed to enable the identification and referral of patients with unmet needs who may significantly benefit from participating in cancer rehabilitation and exercise programming, similar to the suggested pathway described by Santa Mina et al.^18^ Following its implementation, we sought to evaluate the program. As such, the objectives of the present study were to (1) examine the effects of the program, (2) examine whether those effects were maintained 3 months following participation in the program, and (3) explore participant experience of the program.

## 2 METHODS

### 2.1 Design

We conducted a mixed methods, prospective cohort study to examine the effects of a multidimensional cancer rehabilitation program in a mixed population of cancer survivors. The evaluation of the program was guided by the Institute for Healthcare Improvement Triple Aim.^19^ This study was approved by the University Health Network (UHN) Research Ethics Board.

### 2.2 Setting

At the Princess Margaret Cancer Centre, the Cancer Rehabilitation and Survivorship (CRS) clinic offers numerous services for adult (18 years or older) cancer survivors. The services offered through the CRS clinic are funded through the cancer center's foundation and are free to participants. The CRS clinic receives referrals from oncologists who identify a physical, functional, or cognitive impairment that may be addressed by rehabilitation. Following a joint initial comprehensive assessment in the CRS clinic with a physiatrist and physiotherapist or occupational therapist, and a discussion with the patient regarding their rehabilitation goals, a care plan is developed that may include registration into an 8‐week exercise and education program. The 8‐week program is delivered in‐person at the Electronic Living Laboratory for Interdisciplinary Cancer Survivorship Research (ELLICSR): Health Wellness and Cancer Survivorship Centre at UHN, and is called Cancer Rehabilitation and Exercise at ELLICSR (CaRE@ELLICSR). The ELLICSR gym contains a variety of aerobic exercise equipment (e.g., treadmill, stationary cycle, elliptical) and resistance training equipment (e.g., resistance bands, free weights, stability balls). All CRS staff delivering the program were trained in motivational interviewing^20^, ^21^ by a certified Motivational Interviewing Network Trainer. CRS staff were instructed to utilize motivational interviewing skills when delivering the program components (e.g., goal setting, education, exercise delivery).

### 2.3 Participants and recruitment

Given that CaRE@ELLICSR is delivered as part of routine care at the cancer center, participation in the research study evaluating the program was optional and not required to participate in the program. Starting in September 2017, newly registered patients in the program were invited to participate in the study and recruitment was ongoing until February 2020. Participants were asked for their written consent to use the data collected as part of their clinical visits for research purposes. In addition, they were asked if they would agree to be contacted to participate in a qualitative interview about their experience in the program.

### 2.4 Intervention: CaRE@ELLICSR

CaRE@ELLICSR was adapted from the Wellness and Exercise for Cancer Survivors Program, which demonstrated clinically relevant improvements in functional outcomes and high participant satisfaction.^22^ Over the course of the study period, CaRE@ELLICSR underwent routine quality improvement initiatives that resulted in additional program adaptations to enhance participant experience (Table 1). Notable changes included the addition of an online platform to prescribe exercise to participants (i.e., Physitrack®), and wearable technology (i.e., Fitbit™) to facilitate adherence to the prescribed exercise intensity and promote motivation for overall physical activity.

**TABLE 1:** Modifications to CaRE@ELLICSR during the study period.

Date	Number of participants receiving modifcation	Modification	Description
March 2018	174	Addition of aerobic exercises	The format of weekly exercise classes was modified from resistance‐only classes to individualized circuits alternating between resistance and aerobic exercise
November 2018	102	Addition of motivational interviewing techniques	Comprehensive training and ongoing supervision on motivational interviewing was provided to all health care providers in the program. Training was provided by a Motivational Interviewing Network of Trainers certified trainer
January 2019	83	Group exercise class and education booster sessions at follow up appointment (T2)	Format of T2 visit was changed from individual appointments with a RKin, to a group exercise class with an individual fitness assessment completed before, during, or after the exercise class. Additionally, a social worker facilitated a 30‐min group discussion about barriers experienced in the past 3 months
January 2019	83	Online exercise prescription delivery	Participants were provided with access to an online application (i.e., Physitrack™) with video, audio, and written instructions for all exercises prescribed. Participants were able to track their adherence and progress and connect with their RKin if needed. RKins could also monitor and adjust participants' exercise prescriptions remotely
April 2019	52	Additional exercise equipment for classes	Various types of aerobic exercise equipment (e.g., elliptical, recumbent bike) were purchased and incorporated into the exercise classes to facilitate the circuit‐based format
May 2019	40	Wearable technology provided to participants	Each participant was offered a Fitbit™ for the duration of the program to track activity and sleep

The CaRE@ELLICSR program is delivered in‐person, once per week over 8 weeks in groups of 8–10 cancer survivors. Each session includes a 1‐h exercise class, followed by a 1.5‐h educational session on topics that were identified through a large needs assessment. In addition to consultation and feedback from the CRS clinical team, the needs assessment included a survey with cancer survivors (*n* = 60) at the center to identify the most relevant topics for self‐management skills teaching, the length of the program, and the duration of the classes. Prior to beginning the program, each participant undergoes an in‐person assessment conducted by a Registered Kinesiologist (RKin). This assessment includes a review of the participant's clinical history, symptom burden, current level of physical activity, and functional capacity, which are used to create an individualized exercise prescription.

Each exercise prescription was orginally developed to target the 2010 American College of Sports Medicine exercise guidelines for cancer survivors.^23^ This was modified during the study period to target the updated 2019 guidelines of a minimum of 90 min per week of moderate to vigorous intensity aerobic exercise (progressing to 150 min) and 2 to 3 days of resistance training (minimum 2 sets of 8–12 repetitions for major muscle groups).^24^ Each exercise prescription is partially completed in‐person, with the remaining dose expected to be completed independently between classes. Participants are provided with resistance bands to take home to facilitate the completion of their resistance exercise prescription. Participants are provided with a workbook that contains information about the program, their detailed exercise prescription with descriptions of the prescribed exercises and a workout log to track their exercise (adapted to Physitrack®), and information to support each week's education class.

Each exercise class is supervised by two RKins, with additional support by a physiotherapist during the first week. During exercise classes, participants perform their individualized exercise prescriptions using a circuit of resistance training and aerobic exercise (adapted from a resistance‐only format). Following the completion of the first resistance exercise, participants are instructed to complete 3 min of aerobic exercise at 50%–80% of their estimated heart rate range using any of the available equipment. Participants are instructed to repeat this until the completion of their prescribed resistance exercises (i.e., 5–7 cycles).

After each exercise session, participants have a 15‐min break and are relocated as a group to an adjacent classroom where educational sessions are conducted. Each 90‐min class is led by clinicians with expertise in the weekly topic. Education topics include: Getting Started—Goal Setting; Eat and Cook for Wellness; Manage Fatigue and Improve Sleep; Managing your Emotions; Be Mindful; Boost Your Brain Health; Find Ways to Connect; and Plan for Your Future. During these classes, the facilitators review the importance of each topic and its relationship to cancer rehabilitation and survivorship, and participants are encouraged to share their experiences and engage with each other. Additionally, the facilitators review several self‐management techniques to manage cancer‐related impairments and promote behavior change, and they also provide an opportunity for participants to set goals related to incorporating these strategies into their routines (Table 2).

**TABLE 2:** Education topics and examples of self‐management strategies.

Session Topic	Session Facilitator(s)	Discussion topics and self‐management strategies
Getting Started—Goal Setting	Occupational therapist or Kinesiologist	Overview of CaRE@ELLICSR Common changes after cancer Tips to eating before and after exercise Stages of behavior change SMART goals (specific, measurable, action, realistic, time bound)
Eat and Cook for Wellness	Registered Dietician and Wellness Chef	Plant‐based foods Whole grains and whole foods Healthy fats Avoiding or limited alcohol Avoiding processed meat Cooking skills
Manage Fatigue and Improve Sleep	Occupational therapist	Tracking fatigue patterns Activity and exercise Ways to get enough sleep and rest Distraction techniques Modified return to work plans
Managing your Emotions	Social worker	Cognitive behavioral thought record Improving emotional wellbeing—STRONG (Sleep; Treating illnesses; Resisting drugs/alcohol; Once a day, do something to build mastery; Nutrition; Exercise) Acceptance, affirmations, and gratitude Reasons you may need more help
Be Mindful	Occupational therapist	Deap breathing Relaxation exercises Medidation STOP (Stop, take a few breaths, Observe your thoughts and emotions, Proceed with a mindful activity)
Boost your Brain Health	Neuropsychologist	External memory aids Reduce noise Getting organized, decluttering, and maintaining routines Repetition and reviewing (repeat aloud, daily diary) Lifestyle (e.g., stress management, exercise, sleep)
Find Ways to Connect	Occupational therapist	Managing relationship changes after cancer (readjusting expectations, setting boundaries, finding new roles and responsibilities, finding new ways to be intimate) Examples of where to connect (community programs and support groups, giving back, online, recreation, professional support)
Plan for your Future		Review of program goals Setting new goals following program completion Cancer and non‐cancer related community resources

### 2.5 Measurement

As part of routine care for those attending the survivorship clinic, participants completed a questionnaire package using a tablet that included measures for cancer‐related symptoms, disability, and physical activity. Participants also completed a fitness assessment conducted by an RKin at baseline (T0), and at 8 (T1) and 20 (T2) weeks. All assessments were completed at ELLICSR. Qualitative interviews were conducted after T2 with a sub‐sample of participants (*n* = 16) in‐person or over the phone.

### 2.6 Outcomes

Demographic and clinical information were obtained by chart review at baseline and from the initial assessment questionnaire. Data included age, sex, ethnicity, marital status, education, employment status, socioeconomic status, primary cancer location, time since diagnosis, comorbidities, treatment status, and reason for referral.

Disability was measured using the 12‐item World Health Organization's Disability Assessment Schedule 2.0 (WHO‐DAS 2.0).^25^, ^26^ Symptom severity was measured using the Edmonton Symptom Assessment Schedule revised (ESAS‐r).^27^ Physical Activity was measured using the Godin‐Shephard Leisure‐Time Physical Activity Questionnaire (GSLTPAQ) Leisure Score Index (LSI).^28^ The 6‐min walk test (6MWT) was used to assess aerobic functional capacity in accordance with the American Thoracic Society protocol.^29^ Upper body muscular strength was assessed with grip strength measured bilaterally with a standard Jamar dynamometer in accordance with the Canadian Society for Exercise Physiology protocol. Particiapnts' weight and height were used to calculate their body mass index.

#### 2.6.1 Program attendance

Class attendance was obtained by chart review and was calculated as a percentage of classes attended out of 8. While participants were encouraged to use a workout log or Physitrack® to record their exercise during the program, these tools were used to assist the RKins leading the classes in reviewing the participant's exercise prescription in a timely manner. This information was not collected for research purposes. In addition, this study did not collect the number of referrals made to the CaRE@ELLICSR program, reasons for refusal to participate in the program, and reasons for missed classes or follow‐up assessments.

#### 2.6.2 Qualitative assessment of participant experience

We employed qualitative research methods, collecting data using in‐depth, 1‐h, semi‐structured interviews to elicit an understanding of participants' experiences in CaRE@ELLICSR. A convenience sample of participants who had completed the 8‐week program and had completed their 3‐month follow‐up assessment were invited to participate in the interviews. Interviewing participants three or more months after completing program provided an opportunity to explore potential factors influencing the maintenance of exercise and other self‐management strategies. Each interview included open‐ended questions along with relevant prompts that inquired about perceptions of various program components, facilitators and barriers related to attendance, and impact of the program on aspects of their daily lives. The semi‐structured nature and iterative process also enabled a flexible interview script in which interesting findings that emerged from one interview could be probed for in subsequent interviews. Following several interviews with participants, we purposively sampled and recruited male participants to ensure the interviews reflected different perspectives prior to reaching saturation of responses and themes. All interviews were recorded and transcribed verbatim prior to analysis.

### 2.7 Statistical and qualitative analysis

Demographic and clinical data are reported using descriptive statistics. Continuous data are reported as means and standard deviations along with median and range and categorical data are reported as frequencies and percentages. Program effects were assessed in accordance with the intention‐to‐treat principle using linear mixed effects models. Models were fitted with the following covariates: age (years), household socioeconomic status (prefer not to answer, <40,000, 40,000–75,000, or >75,000), cancer stage (I/II or III/IV), number of comorbidities (0–1, 2–3, or >3), years since diagnosis (0–1 years, >1–2 years, >2–5 years, >5 years), and program status (complete or dropout). The proportion of participants with moderate or high disability (WHODAS 2.0) was also examined across time points using Generalized Estimating Equations procedures. All statistical analyses were conducted using SAS Version 9.3. Statistical significance was considered as *p* < 0.05.

Interview transcripts were analyzed for main themes using an inductive approach to thematic analysis.^30^ All transcripts underwent an initial round of coding in which each transcript was coded line‐by‐line to generate a broad sense of the data. Two members of our team with expertise in qualitative research (VT and CP), developed an initial coding framework by reading and analyzing the same three transcripts. This coding framework was then applied to and refined as analysis of the remaining transcripts took place. This process resulted in the development of a codebook, consisting of code names, definitions, example data, and analytical summaries. The codebook was applied in a second round of coding to ensure consistency of analysis across the interviews. Once all transcripts were coded, final themes were assessed. Memoing was conducted throughout the process to keep track of initial perceptions of the data and interesting findings that would be probed for in subsequent interviews.^31^

## 3 RESULTS

### 3.1 Participants

**FIGURE 1:**
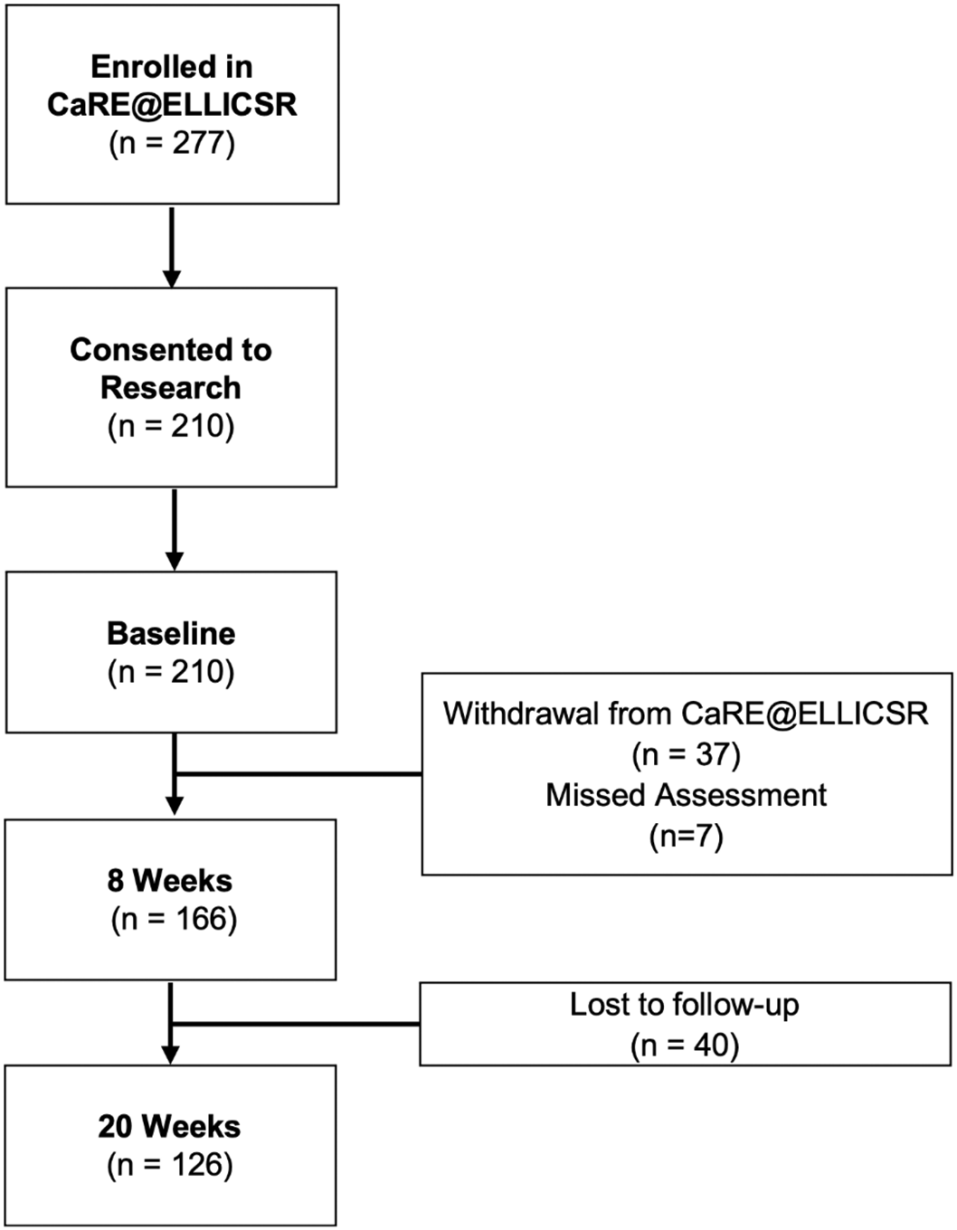
Participant flow and attendance.

During the study period, 277 cancer survivors were registered into the CaRE@ELLICSR program, and 210 consented to participate in the research study (76%). The numbers of participants completing each assessment in the program are presented in Figure 1.

Demographic and clinical characteristics of the study participants are displayed in Table 3. Briefly, the mean age of participants was 55 years and participants were mostly female (78%), white/Caucasian (55%), and married or in a common‐law relationship (57%). The most common cancer types were breast cancer (50%), gynecologic cancer (21%), and lymphoma and myeloma (12%). At the start of program participation, 56% reported having completed primary treatment, and 33% reported currently receiving endocrine therapy. The median number of classes attended by participants was 7 (interquartile range = 6–8).

**TABLE 3:** Participant demographic data (*N* = 210).

Characteristic	Participants
Age (years), median (range)	55 (22–88)
Sex, *n* (%)	
Female	163 (78)
Male	47 (22)
Ethnicity, *n* (%)	
White/Caucasian	115 (57)
South Asian	28 (14)
East Asian	17 (8)
Black/Afro‐Caribbean/African	11 (5)
Latino/Hispanic	9 (4)
Other	23 (11)
Not reported	7
Marital Status, *n* (%)	
Married/Common‐law	116 (57)
Single	47 (23)
Divorced/Widowed/Other	40 (20)
Not reported	7
Education, *n* (%)	
High school or less	20 (10)
Some university or college	32 (16)
Finished university or college	106 (53)
Other	41 (21)
Missing	11
Socioeconomic status, *n* (%)	
<$75,000	60 (30)
>$75,000	69 (35)
Prefer not to answer	71 (36)
Not reported	10
Employment, *n* (%)	
Not employed/on disability/retired	118 (62)
Employed	73 (38)
Not reported	19
Cancer type, *n* (%)	
Breast	105 (50)
Lymphoma and myeloma	26 (12)
Gynecologic	21 (10)
Genitourinary	12 (6)
Head and neck	10 (5)
Gastrointestinal	9 (4)
Lung	7 (3)
Leukemia	6 (3)
Other^a^	14 (7)
Cancer stage, *n* (%)	
I	59 (30)
II	61 (31)
III	39 (20)
IV	36 (18)
Missing or unavailable	15
Current treatment, *n* (%)	
None	118 (56)
Endocrine therapy	70 (33)
Targeted therapy	11 (5)
Chemotherapy	6 (3)
Immunotherapy	4 (2)
Radiotherapy	1 (1)
Reason for referral, *n* (%)^b^	
Fatigue	149 (71)
Therapeutic exercise prescription/deconditioning	105 (50)
Neurocognitive	88 (42)
Psychosocial support	81 (39)
Musculoskeletal	66 (31)
Diet and nutrition	41 (20)
Return to work	19 (9)
Insominia	12 (6)
Pain	7 (3)
Time from diagnosis to initial assessment (years), median (range)	1.26 (0.16–15.4)

^a^ Includes central nervous system and eye, endocrine, melanoma, and sarcoma.

^b^ Participants can be referred for multiple reasons.

### 3.2 Outcomes

#### 3.2.1 Quantitative findings

**FIGURE 2:**
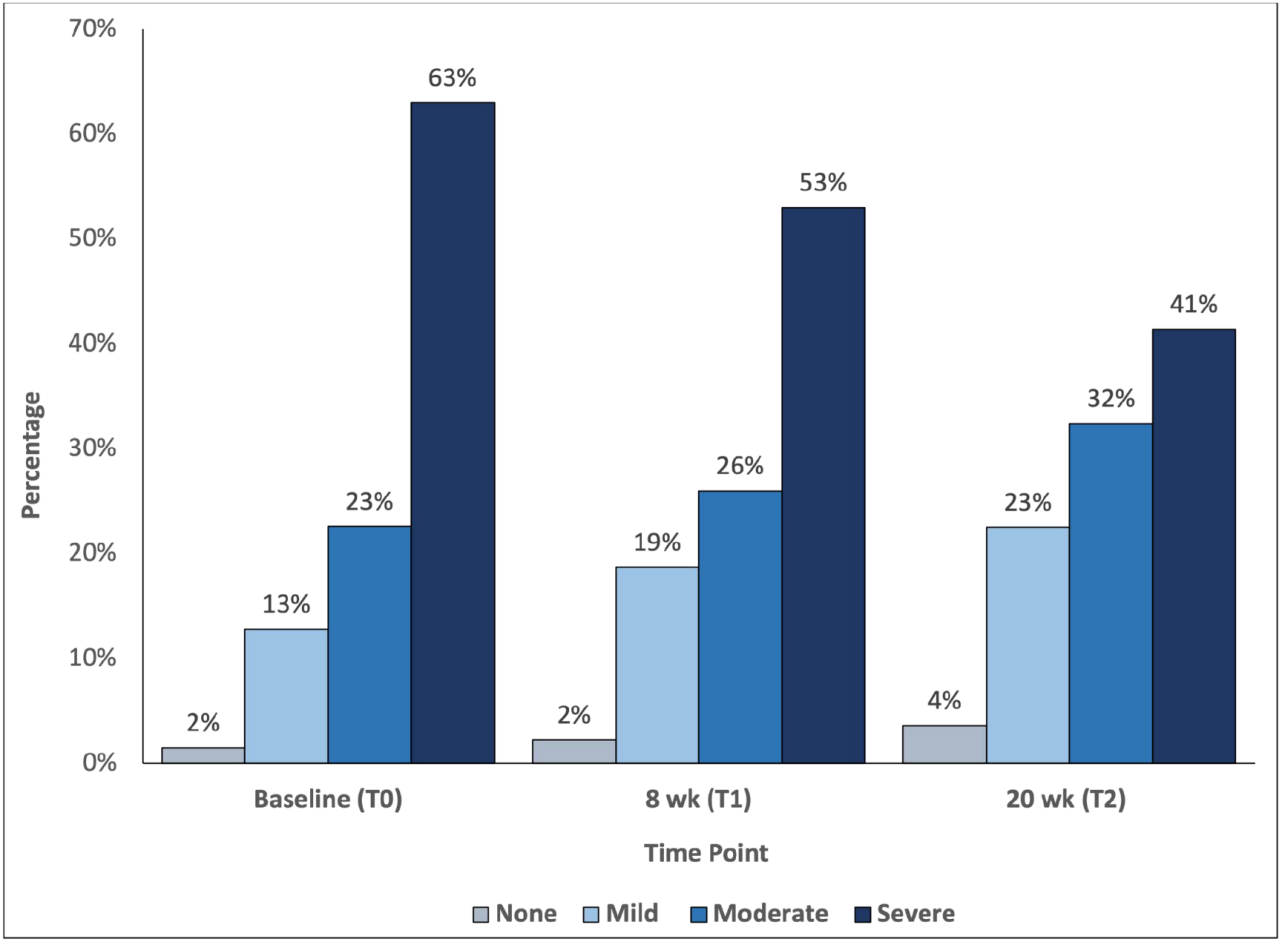
Proportion of participants reporting each WHO‐DAS category at each study time point. Percentages are displayed for each time point in blue‐gray (no disability), light blue (mild disability), blue (moderate disability), and navy blue (severe disability).

Figure 2 displays the proportion of participants who reported no disability, mild disability, moderate disability, or severe disability via the WHO‐DAS. From baseline (T0) to 20 weeks (T2), the proportion of participants reporting moderate to severe levels of disability significantly decreased, where the likelihood of reporting none or mild disability was 2.18 times higher at T2 when compared with baseline (*p* = 0.031).

Table 4 presents means and standard errors for each outcome over time. From baseline (T0) to the end of the 8‐week program (T1), statistically significant changes were observed for the WHO‐DAS (−1.81, 95% confidence interval (CI): −2.59 − (−1.04)), ESAS tiredness (−0.51, 95% CI: −0.84 − (−0.19)), ESAS anxiety (−0.45, 95% CI: −0.76 − (−0.14)), ESAS depression (−0.41, 95% CI: −0.73 − (−0.10)), ESAS wellbeing (−0.39, 95% CI: −0.77 − (−0.02)), total LSI (+13.3, 95% CI: 11.1–18.8) and moderate to strenuous LSI (+13.2, 95% CI: 9.88–16.5) via the GSLTPAQ, 6MWT (+37.4 m, 95% CI: 29.1–45.7), and grip strength (+4.78 kg, 95% CI: 3.55–6.02).

**TABLE 4:** Estimates of outcomes measures.

Outcome Measure	Time Point	*N*	Mean (SE)	95% CI	Difference in the Estimates (95% CI)	*p*‐Value
WHO‐DAS	T0	195	14.5 (0.87)	12.7–16.2	–	–
	T1	134	12.6 (0.86)	10.9–14.3	−1.81 (−2.59 − (−1.04))	**<0.001**
	T2	111	12.0 (0.88)	10.3–13.7	−2.46 (−3.42 − (−1.50))	**<0.001**
ESAS‐r Measures						
Pain	T0	203	3.15 (0.24)	2.67–3.63	–	–
	T1	141	3.09 (0.26)	2.58–3.61	−0.06 (−0.39–0.27)	0.714
	T2	113	2.92 (0.26)	2.40–3.45	−0.23 (−0.59–0.14)	0.217
Tiredness	T0	203	4.69 (0.25)	4.19–5.19	–	–
	T1	141	4.17 (0.26)	3.65–4.70	−0.51 (−0.84 − (−0.19))	**0.002**
	T2	113	4.12 (0.29)	3.56–4.69	−0.56 (−0.99 − (−0.13))	**0.012**
Drowsiness	T0	203	3.05 (0.26)	2.54–3.56	–	–
	T1	141	3.08 (0.27)	2.55–3.61	−0.03 (−0.33–0.38)	0.178
	T2	113	2.94 (0.28)	2.39–3.50	−0.11 (−0.58–0.36)	0.238
Depression	T0	202	3.06 (0.28)	2.51–3.60	–	–
	T1	141	2.65 (0.28)	2.09–3.20	−0.41 (−0.73 − (−0.10))	**0.012**
	T2	113	2.87 (0.28)	2.31–3.43	−0.19 (−0.56–0.18)	0.315
Anxiety	T0	202	3.52 (0.28)	2.95–4.07	–	–
	T1	141	3.07 (0.28)	2.51–3.63	−0.45 (−0.76 − (−0.14))	**0.005**
	T2	113	3.03 (0.29)	2.46–3.61	−0.48 (−0.85 − (−0.11))	**0.010**
Wellbeing	T0	202	4.41 (0.24)	3.93–4.89	–	–
	T1	141	4.01 (0.25)	3.52–4.51	−0.39 (−0.77 − (−0.02))	**0.038**
	T2	113	3.97 (0.26)	3.93–4.89	−0.44 (−0.85 − (−0.03))	**0.034**
GODIN						
Total LSI	T0	210	16.7 (1.91)	13.0–20.5	–	–
	T1	134	31.7 (2.49)	26.8–36.6	14.9 (11.1–18.8)	**<0.001**
	T2	115	24.0 (2.41)	19.2–28.7	7.21 (3.28–11.1)	**<0.001**
Moderate to strenuous LSI	T0	210	6.85 (1.42)	4.04–9.70	–	–
	T1	135	20.0 (2.06)	16.0–24.1	13.2 (9.88–16.5)	**<0.001**
	T2	115	13.2 (1.89)	9.50–16.7	6.39 (3.37–9.40)	**<0.001**
Six minute Walk Test (Meters)	T0	203	442 (10.5)	422–463	–	–
	T1	155	480 (10.1)	460–500	37.4 (29.1–45.7)	**<0.001**
	T2	106	480 (10.5)	460–501	37.8 (28.5–47.2)	**<0.001**
Grip Strength (kg)	T0	210	50.9 (2.05)	46.8–54.9	–	–
	T1	162	55.6 (2.08)	51.5–59.7	4.78 (3.55–6.02)	**<0.001**
	T2	118	56.6 (2.14)	52.3–60.7	5.69 (4.16–7.24)	**<0.001**
Body Mass Index (kg/m^2^)	T0	196	28.2 (0.76)	26.7–29.7	–	–
	T1	153	28.3 (0.76)	26.8–29.8	0.10 (−0.10–0.31)	0.325
	T2	115	28.3 (0.75)	26.8–29.8	0.08 (−0.15–0.32)	0.474

Bold indicates statistical significance (p < 0.05).

From baseline (T0) to 20‐weeks (T2), statistically significant changes in outcome measures were also observed for the WHO‐DAS (−2.46, 95% CI: −3.42 − (−1.50)), ESAS tiredness (−0.56, 95% CI: −0.99 − (−0.13)), ESAS anxiety (−0.48, 95% CI: −0.76 − (−0.14)), ESAS wellbeing (−0.44, 95% CI: −0.85 − (−0.03)), total LSI (+7.33, 95% CI: 3.28–11.1) and moderate to strenuous LSI (+6.39, 95% CI: 3.37–9.40) via the GSLTPAQ, 6MWT (+37.8 m, 95% CI: 28.5–47.2), and grip strength (+5.69 kg, 95% CI: 4.16–7.24). No other statistically significant changes were observed in any of the other measured variables during the 8‐week (T1) or 20‐week (T2) timepoints.

#### 3.2.2 Qualitative findings

Twenty‐six participants were invited to be interviewed, and 16 agreed. Most interviewees were female (56%) and married (69%). Interview participants were treated for breast cancer (44%), genitourinary cancer (44%), or gynecological cancer (12%). The interviews revealed three key themes described in Table 5 along with representative quotes. Briefly, learning to prioritize exercise and healthy living as actionable strategies for rehabilitation and the improvements in physical and functional wellbeing observed through the quantitative surveys and fitness assessments, were often connected back to an improved sense of empowerment and control over their recovery in the qualitative interviews (theme 1, empowerment and control). Furthermore, the ability to meaningfully and successfully participate in the program and adhere to exercise and other program components was often connected back to having access to appropriate supports. Participants emphasized the importance of the supervision from the multidisciplinary staff delivering the program, as well as the benefits of exercising and socializing with other cancer survivors who had shared experiences (theme 2, supervision and internal program support). Participants also highlighted the importance of having access to quality supports outside the center to maintain the learned exercise and other self‐management stratagies at home, both during and after the program (theme 3, external program support).

**TABLE 5:** Qualitative themes and representative quotes.

Theme	Description	Example Quote
Empowerment and Control	Participants reported a change in how they perceived their lives after their cancer diagnosis and frequently described changes related to their identity as a cancer survivor. Through exercise and other self‐management strategies such as tips to managing fatigue, brain fog, and health eating, participants reported improvements in their condition which altered their beliefs of what it meant to be a cancer survivor and their perceptions on the importance of these behaviors. Participants expressed that their participation enabled them to be more optimistic about their future and in control of their decisions and capabilities post‐treatment	“I [am less] focused on the disease and seeing myself as cycling through the same thing of like, ‘why did this happen to me.’ Now it's more like looking ahead. […] The exercise is good and now it's a priority for me. This is what I have to do to feel good. Same thing with nutrition—I'm more self‐aware at prioritizing my wellbeing.”—Female, gynecologic cancer. “I feel more confident, and I know what's going on and how to handle it. It's not like my life is perfect now, but it seems like I'm more in control of what's going on. I know myself more. […] The [program] has given me more opportunities to be more optimistic about my life.”—Female, gynecologic cancer
Supervision and Internal Program Support	Participants highlighted the benefits of having supervised and individualized exercise prescriptions from RKins with expertise and experience in oncology. They indicated that the supervised and individualized nature of the classes provided them with greater confidence and motivation to incorporate exercise into their lives. However, some participants indicated that this supportive environment was difficult to recreate at home, which was a barrier to exercising independently. Participants underscored the ability to discuss important issues with other cancer survivors. They indicated that this may not always be easy to do with family and friends, and that the classes felt liberating and offered a more solutions‐based discussion. They expressed that they were able to be more honest about their experiences and challenges. Participants indicated that the education classes provided them with a sense of validation and normalized their experiences post‐treatment, which allowed them to focus on how to better manage their cancer‐related impairments	“Coming here and listening to lectures helped me get into a routine and after I get into a routine it's just a matter of maintaining it which I've been doing successfully. But if you just give me some books and say, ‘go home and do this’ I might not be able to. I might not even get started.”—Male, genitourinary cancer “There was an effort to encourage us to integrate the information into our lives but it's so challenging because you go [to ELLICSR] and it's an artificial world in a way. It's a nice little bubble. And when you leave, you can't take the ELLICSR people with you when you go back home. […] It's hard for it to carry over, to actually do the journaling, or to cook the recipes or trying to find the time for those things.”—Female, breast cancer
External Program Support	Participants emphasized the importance of having supports outside the program to facilitate adherence to exercise and other self‐management strategies. Support from family included driving participants to the classes, accompanying participants during prescribed walking, and assisting participants in implementing recipes learned during the diet and nutrition class. Alternatively, unsupportive relationships hindered regular exercise at home. For instance, some participants indicated that friends and family held a misconceived dichotomy in which having completed treatment or being in remission represented ‘normal’ functioning. Having supportive employers who enabled a gradual return to work allowed participants to focus on their recovery. However, some participants reported having to return to work before they were physically and mentally ready due to pressures from their employer or financial challenges. As a result, some participants indicated that it was challenging to find time to maintain exercise or other self‐management strategies after the program	“Your family expects you to do this, to do that. And I have to talk to them because of the chemo fog, I easily forget something. Don't blame me on that. It's stressful. They expect you to be normal.”—Female, breast cancer “My boss is a very nice, very understanding person, and he said, ‘no problem, whatever time you need, just take it off and do what you need to do.’”—Male, genitourinary cancer

## 4 DISCUSSION

From a public health perspective, disability is considered as important as mortality^32^ and cancer‐related disability is a prevalent adverse effect of cancer and its treatments.^1^ Oncology clinical practice guidelines commonly recognize the need and benefits of rehabilitation and exercise and recommend these services for people living with and beyond cancer.^7^ However, there is a gap in the implementation of these guidelines within the delivery of cancer care, which is reflected in the limited number of rehabilitation and exercise programs available for cancer survivors and the low rates of referrals to existing services.^33^, ^34^, ^35^ While the evidence from systematic reviews and meta‐analyses clearly demonstrate the benefits of exercise interventions on managing cancer‐related impairments,^10^, ^12^ the inadequate number of exercise and rehabilitation programs embedded into clinical care limits our understanding of their effects in real‐world settings.

This study describes the effects of an 8‐week, group‐based, multidimensional cancer rehabilitation and exercise program for people with cancer delivered as part of routine care. Participants in the program achieved significant improvements in disability, physical activity, aerobic functional capacity, upper body muscular strength, as well as several cancer‐related symptoms. These findings are consistent with evaluations of real‐world community‐based exercise oncology programs in Canada.^36^, ^37^, ^38^ Further, most of these outcomes were maintained 3 months after completing the CaRE@ELLICSR program. In fact, participants were approximately twice as likely to report mild or no disability after completing the program than at baseline. Additionally, the average increase of 37 metere via the 6MWT and 5.7 kg via grip strength observed in this study are considered clinically meaningful.^39^, ^40^ Given that cancer and its treatments can negatively impact functional capacity, improvements in these outcomes are essential for cancer survivors.^41^

Participants in the current study also reported positive experiences with the program and highlighted its importance to their recovery and wellbeing. Results from the qualitative interviews indicate that attendance to the classes was in part facilitated by the group environment, including the ability for participants to share their experiences with each other and normalize many of the cancer‐related impairments they were experiencing. This is consistent with a systematic review describing the social benefits of exercise‐based rehabilitation.^42^ Further, participants in the program were able to maintain improvements in their level of physical activity after the program, and they highlighted the benefits of the program on their ability to take control of their recovery and wellbeing. This reflects findings from a systematic review, where the most common facilitators of exercise reported by cancer survivors were gaining a sense of control over their health and managing emotions and mental well‐being.^43^ Notably, participants in the program also underscored the importance of gaining confidence in exercise and other self‐management strategies and having support from family and employers to adhere to these behaviors during and after the program.

This study demonstrates the real‐world impact of overcoming common organizational barriers to deliver exercise and rehabilitation programming as part of routine care. However, it is important to highlight that CaRE@ELLICSR is delivered in a large urban cancer center that is highly resourced and supported through a well‐funded cancer foundation. As such, there may be significant challenges translating these findings to under‐resourced and under‐funded settings. These settings may need to adapt the in‐person model utilized by CaRE@ELLICSR to facilitate the implementation and delivery of critical multidimensional rehabilitation programming to their patient population. Distance‐based eHealth interventions have been suggested as one method to reduce barriers to accessing and providing rehabilitation.^44^, ^45^ Recognizing this challenge and opportunity, our team adapted CaRE@ELLICSR to develop the 8‐week CaRE@Home program, which consists of weekly remote check‐ins for exercise and behavior change support, as well as e‐modules providing interactive education to promote self‐management skills. Preliminary results indicate that the CaRE@Home program is feasible, acceptable, and may decrease disability,^46^ highlighting the potential to translate the effects of CaRE@ELLICSR to settings that do not have sufficient resources and funding.

A limitation of this study is the lack of a control group. In the absence of a control group, it is possible that other factors could have contributed to the observed results (e.g., natural course of recovery, participation in additional rehabilitation services and programs within and outside of the hospital). Notably, the qualitative findings support the results of the quantitative outcomes, suggesting that participation in the program had a positive impact on participants' recovery and wellbeing. However, all participants interviewed had completed the program and the results do not include perspectives from participants who were unable to complete the program. In addition, about 80% of the participants in this study were female and half had breast cancer, potentially biasing the generalizability of the results. This may have been due to differences in referral patterns among physicians across disease site clinics at the cancer center (e.g., differences in screening for rehabilitation needs), as well as differences among patients in the likelihood to discuss rehabilitation services with their health care provider. The CRS clinic has a longstanding relationship with the breast clinic at the center and many oncologists were familiar with the clinic's services compared to providers working in other disease sites. Information on referral patterns to the CaRE@ELLICSR program will clarify the proportion and representativeness of participants referred and enrolled to the program, as well as reasons for non‐enrollment and participation. As such, the CRS clinic has developed a process for tracking this information through the electronic medical record system and internal databases which will inform ongoing quality improvement initiatives. Future studies would benefit from embedding an implementation science framework in the design of a comprehensive program evaluation. An improved understanding of a program's reach across the cancer center, barriers and enablers to adoption and sustainability, and costs of delivering the program would inform the selection of implementation strategies to embed these programs as part of routine clinical care.^47^

## 5 CONCLUSION

In conclusion, the findings of this study provide important evidence on the health‐related benefits of a multidimensional cancer rehabilitation program, CaRE@ELLICSR, for cancer survivors delivered as part of routine care. This study provides further evidence supporting the integration of cancer rehabilitation and exercise programming within routine cancer care. CaRE@ELLICSR can serve as a model for the delivery of similar programs around the world.

## AUTHOR CONTRIBUTIONS

**Christian J. Lopez:** Data curation (equal); formal analysis (supporting); investigation (equal); visualization (equal); writing – original draft (lead); writing – review and editing (equal). **Daniel Santa Mina:** Conceptualization (equal); formal analysis (supporting); investigation (equal); methodology (equal); supervision (supporting); visualization (supporting); writing – original draft (equal); writing – review and editing (equal). **Victoria Tan:** Data curation (equal); formal analysis (supporting); investigation (supporting); methodology (supporting); writing – review and editing (equal). **Manjula Maganti:** Data curation (lead); formal analysis (lead); visualization (equal); writing – review and editing (equal). **Cheryl Pritlove:** Data curation (supporting); formal analysis (supporting); methodology (supporting); writing – review and editing (equal). **Lori J. Bernstein:** Conceptualization (supporting); supervision (supporting); writing – review and editing (equal). **David M. Langelier:** Supervision (supporting); writing – review and editing (equal). **Eugene Chang:** Conceptualization (supporting); supervision (supporting); writing – review and editing (equal). **Jennifer M. Jones:** Conceptualization (equal); formal analysis (supporting); funding acquisition (lead); investigation (equal); methodology (equal); project administration (lead); supervision (lead); visualization (supporting); writing – original draft (equal); writing – review and editing (equal).

## FUNDING INFORMATION

This study was supported through internal funding provided by the Princess Margaret Cancer Foundation and the Butterfield/Drew Chair in Cancer Survivorship Research.

## CONFLICT OF INTEREST STATEMENT

None.

## ETHICS STATEMENT

This study was reviewed and approved by the University Health Network Research Ethics Board (REB# 17‐5218).

## PARTICIPANT CONSENT

All patients provided written consent for their data to be used for this analysis.

## Data Availability

Due to REB restrictions, we are not permitted to upload data to a public data repository. However, we can make data available upon request with a data transfer agreement.
